# LowCOST‐HSQC Variants for Fast‐Pulsing High *ω*_1_‐Resolved 2D Experiments

**DOI:** 10.1002/mrc.70102

**Published:** 2026-04-16

**Authors:** David Schulze‐Sünninghausen, Johanna Becker, Martin R. M. Koos, Burkhard Luy

**Affiliations:** ^1^ Bruker BioSpin GmbH & Co. KG Ettlingen Germany; ^2^ Currenta GmbH & Co. OHG, Analytik Leverkusen Germany; ^3^ Pfizer Incorporated Groton Connecticut USA; ^4^ Institute of Organic Chemistry and Institute for Biological Interfaces 4—Magnetic Resonance Karlsruhe Institute of Technology (KIT) Eggenstein‐Leopoldshafen Germany

**Keywords:** ASAP, fast‐pulsing 2D NMR, HSQC, LowCOST, TIG‐BIRD, ZIP

## Abstract

The LowCOST‐HSQC is a sensitivity‐enhanced HSQC version that retains unused proton polarization for subsequent scans in correlations to low natural abundance nuclei like 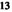
C or 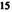
N. Together with fast polarization distribution via isotropic mixing, it allows the acquisition of fast‐pulsing 2D experiments. We give a detailed introduction and comparison of three possible INEPT‐type transfer elements for the LowCOST approach—the original LowCOST, the ZIP, and a specific TIG‐BIRD element—and evaluate various variants of the LowCOST‐HSQC.

## Graphical abstract

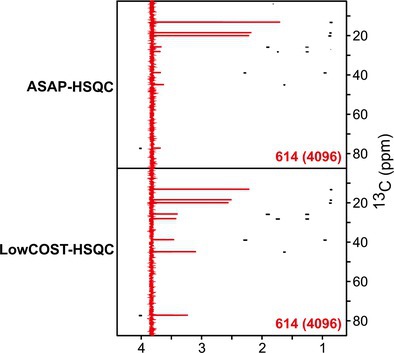

## 1 Introduction

Fast‐pulsing 2D experiments have demonstrated their incredible performance. Starting from the revolution in protein NMR, where the FAST and SO‐FAST experiments [1, 2, 3] as well as the many different BEST experiments [4, 5, 6] lead to a vast reduction in measurement times, Ernst‐angle excitation [7, 8, 9] and fast TOCSY‐type equilibration [10, 11] in 2D heteronuclear correlation experiments have dramatically increased the possibilities also in small‐molecule NMR. The best one‐bond correlation experiment with acquisition times down to a couple of seconds is the ASAP‐HSQC [12, 13] and its variants [13, 14, 15, 16]. The approach also allows the acquisition of HSQCs with a $\omega_{1}$ resolution down to 0.5 Hz in less than ten minutes [13]. However, sensitivity enhancement as a well‐known strategy to combine echo/antiecho detection schemes with utmost sensitivity is not compatible with the approach. Furthermore, a detailed analysis revealed that unused polarization is in the transverse plane during $t_{1}$ increments and evolves homonuclear couplings, thereby reducing the accessible reservoir polarization significantly. On the search for a fast‐pulsing HSQC variant tolerating sensitivity enhancement, the LowCOST‐HSQC [17] and the ZIP‐HSQC [18, 19] have been developed independently, leading to sequences following the same underlying principle that differ only in the specific extension of the INEPT‐type forward‐transfer element.

In the following, we will first compare the two transfer elements and a third one resembling a TIG‐BIRD implementation [20, 21], before we introduce several extensions to the sequence. We then provide a constant‐time version, a BIRD 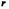 variant with multiplicity editing and decoupling of long‐range heteronuclear couplings in the indirect dimension, and an HSQC‐TOCSY‐type experiment. All sequences are enhanced by broadband‐shaped pulses and demonstrated on small‐molecule examples.

## 2 Results and Discussion

Conventional HMQC‐ and HSQC‐type experiments detect 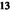
C‐bound proton magnetization, but effectively dephase all unused 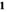
H magnetization not directly bound to 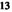
C nuclei. As a consequence, only $T_{1}$ relaxation can build up polarization for the next scan, leading inevitably to long recovery delays $d_{r}$ between scans. The ALSOFAST and ASAP approaches [9, 10, 12, 13] for HMQC/HSQC experiments retain the unused magnetization and allow a much faster recovery via Ernst angle–type excitation and isotropic mixing based redistribution of polarization. In this way, experiment times are reduced dramatically, allowing acquisitions of 2D experiments in less than three seconds or the recording of highly $\omega_{1}$‐resolved 2D‐HSQC spectra on the order of minutes. Particularly, the ASAP‐HSQC provides short, high‐quality spectra with enhanced sensitivity compared with conventional HSQC spectra for a given measurement time. However, for experiments with many increments the sensitivity of spectra not always reaches the desired improvement.

The known downside of the ASAP‐HSQC approach and the cause of signal reduction at long acquisition times in the indirect dimension is the orientation of unused magnetization in the transverse plane during the $t_{1}$‐evolution period. If the acquisition time in the indirect dimension is chosen to be long, 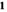
H– 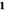
H couplings may evolve, converting spin polarization into coherence unaccessible in following scans. This problem also persists in protein NMR, where the water needs to be controlled to maximize sensitivity in subsequent scans of exchanging amide protons.

One way to solve this issue in protein NMR was developed in the COST‐HSQC (cooling overall spin temperature–HSQC) [22]: Using selective pulses, magnetization is prepared in such a way, that 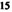
N‐coupled protons polarization is transferred to heteronuclear antiphase magnetization ( $I_{z}\to 2I_{z}S_{x}$), while heteronuclear uncoupled polarization is reoriented along z ( $I_{z}\to I_{z}$). With this trick, not only unused spin polarization is safely stored during $t_{1}$, but also sensitivity enhancement can be applied, which will result in uncoupled magnetization being repositioned along z during acquisition, as the homonuclear part of the sensitivity enhancement sequence represents a perfect echo [23] with inherent planar mixing Hamiltonian. While the original idea is to enhance sensitivity via chemical exchange of amide protons with already polarized water, it may also serve in other ways as a polarization reservoir for the next scan.

It would be good to have a COST‐like approach available for small‐molecule NMR. But in most applications with natural abundance samples, band‐selective treatment using shaped pulses is not possible. The obvious choice, instead, would be the application of bilinear rotations like the BIRD [24], TANGO [25], and BANGO [26] elements with their more complex extensions [27, 28], which treat directly 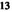
C‐bound spins different from other protons. In the LowCOST‐HSQC [17] and in the ZIP‐HSQC [18], two different elements have been proposed that lead to the desired transfer. While Ernst angle–type excitation is not compatible with the approach, ASAP‐type isotropic mixing for fast polarization redistribution is possible and can be combined with sensitivity enhancement. Next to the two sequences, a third transfer element exists (mentioned already in [19]), as the transfer problem resembles that of the TIG‐BIRD approach [21] or a BIG‐BIRD element [20] with a subsequent INEPT step.

### 2.1 Comparison of Forward‐Transfer Elements

**FIGURE 1:**
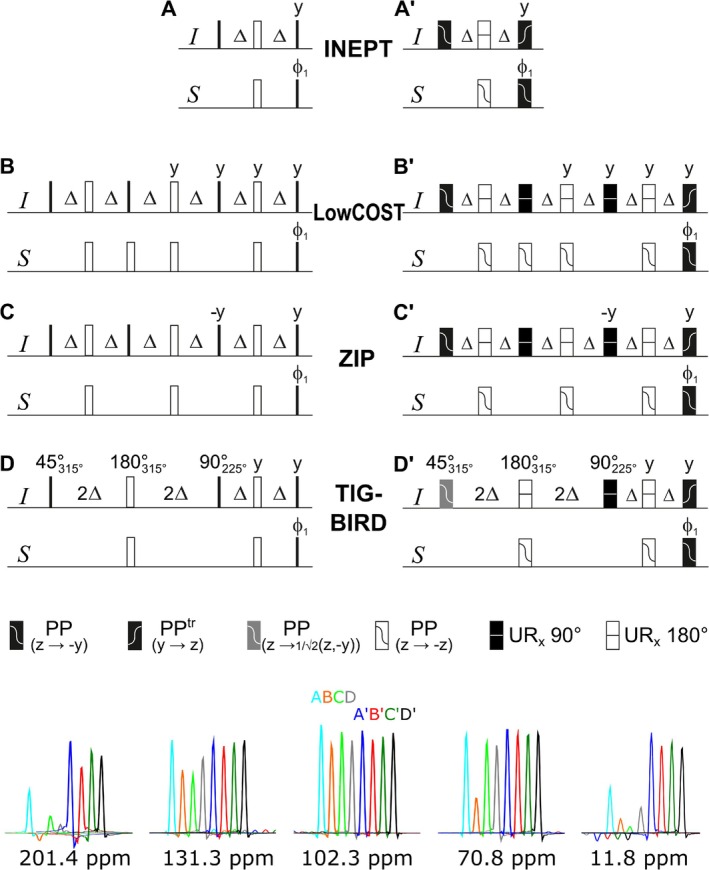
INEPT (A,A^*′*^) and three versions of forward transfer into $2I_{z}S_{y}$ antiphase for heteronuclear coupled spins that at the same time align uncoupled spins for storage as $I_{z}$ polarization during $t_{1}$ delays: the original LowCOST transfer element (B,B^*′*^), the ZIP element (C,C^*′*^), and the corresponding TIG‐BIRD sequence (D,D^*′*^). Sequences indicated with simple letters are hard pulse versions, while letters with a prime mark the corresponding versions with broadband‐shaped pulses. The delay is set to Δ=1/(4J) with a nominal one‐bond coupling J. 90° hard pulses are indicated by solid bars, and open bars refer to 180° hard pulses. Phases are along x unless indexed otherwise. The pictograms for shaped pulses are explained below the sequences, where PP corresponds to point‐to‐point pulses with start and final orientations of spin components as mentioned in parentheses and UR represents universal rotation pulses with the corresponding rotation angle in degrees. All pulses are applied along x unless indicated otherwise. Pulses indicated in degrees with subscript phases in degrees are used if phases differing from Cartesian axes are required. Actual pulse shapes used for experiments have been derived from optimal control theory–based optimizations. For PP( z→−y) and PP( y→z) pulses as well as UR 180( 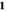
H) and PP( z→ ‐z)( 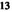
C), the individual pulse shapes of J‐compensated BEBE 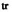 and BUBI pulse sandwiches [43] are applied. The 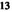
C UR 180° pulse shape is constructed from two corresponding PP( z→−y) shapes following the construction principle [45]. The pulse with an effective flip angle of 45° has been developed for INADEQUATE applications and will be published elsewhere [46]. Resulting intensities in LowCOST‐HSQC experiments with corresponding forward‐transfer elements are shown for a number of cross peaks with 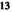
C chemical shifts as indicated. An approximately 100‐mM sample of hydroquinidine and acetaldehyde dissolved in DMSO‐ $d_{6}$ was used on a 600‐MHz spectrometer.

The three different forward‐transfer pulse sequence elements are shown together with a conventional INEPT in Figure 1 in hard pulse (A–D) and shaped pulse variants (A*′*–D*′*). While the conventional INEPT has a single refocused delay of duration 2Δ=1/(2J), where the nominal coupling constant is typically set to an intermediate value of J=145 Hz. All three bilinear rotation type transfer elements require coupling evolution periods with an overall duration of 6Δ. However, the sequences apply four (LowCOST), three (ZIP), and two (TIG‐BIRD) 180° hard pulses on the heteronucleus, which are known to have a severe offset dependence. It is therefore hardly surprising that the evaluation of corresponding hard pulse sequences shows dramatic losses at larger offsets for all three implementations with particular losses for the LowCOST variant. It is therefore an obvious choice to replace hard pulses by offset‐compensated broadband pulse shapes. Using optimal control theory, a large multitude of such shaped pulses has been developed over the last two decades [29, 30, 31, 32, 33, 34, 35, 36, 37, 38, 39, 40, 41, 42], with the BUBI pulse sandwich sticking out, as it is compensated for J‐coupling evolution during simultaneously applied shapes [43, 44]. The shaped pulse sequences with pictograms are given in Figure 1 together with specific pulse shapes stated in the figure caption. With the application of broadband pulses with compensated 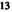
C and 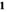
H frequency ranges of 37.5 and 10 kHz, respectively, the transfer efficiency of all three elements changes significantly, leading to essentially identical and highly improved performance. Consequently, any of the shaped pulse elements may be used in the experiment variants that will be introduced in the following paragraphs, and a placeholder is put for any of the forward‐transfer element.

It should be noted that also homonuclear coupling evolution will have an impact on spectral quality, in particular for large proton spin systems with a large number of couplings involved. The best sequence with this aspect is the original INEPT, as the overall duration of the sequence is the shortest with magnetization in the transverse plane for 2 Δ. The 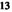
C‐coupled 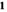
H spins will evolve for 6 Δ in the LowCOST and ZIP case and therefore lead to reduced spectral intensity on the one hand and to more significant virtual, H2BC‐type peaks on the other hand. The TIG‐BIRD sequence excites the first part by 45°, leading only partially to 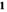
H, 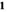
H coupling evolution and to signal reduction and artifact peak intensities that lie in an intermediate range between INEPT on one side and LowCOST and ZIP sequences on the other side.

Next to homonuclear coupling evolution, also mismatch in the large heteronuclear coupling constant relative to the nominal coupling constant used for defining the delay Δ will affect LowCOST, ZIP, and TIG‐BIRD elements stronger than the conventional INEPT element. However, in all examples acquired, we found that the loss connected with this effect is minor compared with the gains in the polarization reservoir during the $t_{1}$ period for highly resolved spectra.

### 2.2 Comparison of LowCOST‐ and ASAP‐HSQC

The ASAP‐HSQC experiment has been studied extensively in both theory and experiment during the last decade [12, 13, 14, 15, 16]. It not only uses Ernst angle–type excitation and isotropic mixing–based fast polarization redistribution to significantly enhance sensitivity but also comes with the caveat that it is generally not compatible with sensitivity‐enhanced back transfer. The LowCOST‐HSQC and the ZIP‐HSQC, instead, provide fast polarization redistribution and sensitivity‐enhanced back transfer but are incompatible with Ernst angle–type excitation. In addition, the already mentioned storage of reservoir magnetization during $t_{1}$ is profoundly different: While ALSOFAST‐ and ASAP‐HSQCs store the reservoir as transverse coherence, LowCOST‐ and ZIP‐HSQCs store the reservoir as polarization along z, effectively avoiding coupling evolution for 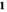
H, 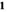
H couplings in the weak coupling limit. Different spin systems and experimental settings will therefore react differently to the fast‐pulsing approaches.

**FIGURE 2:**
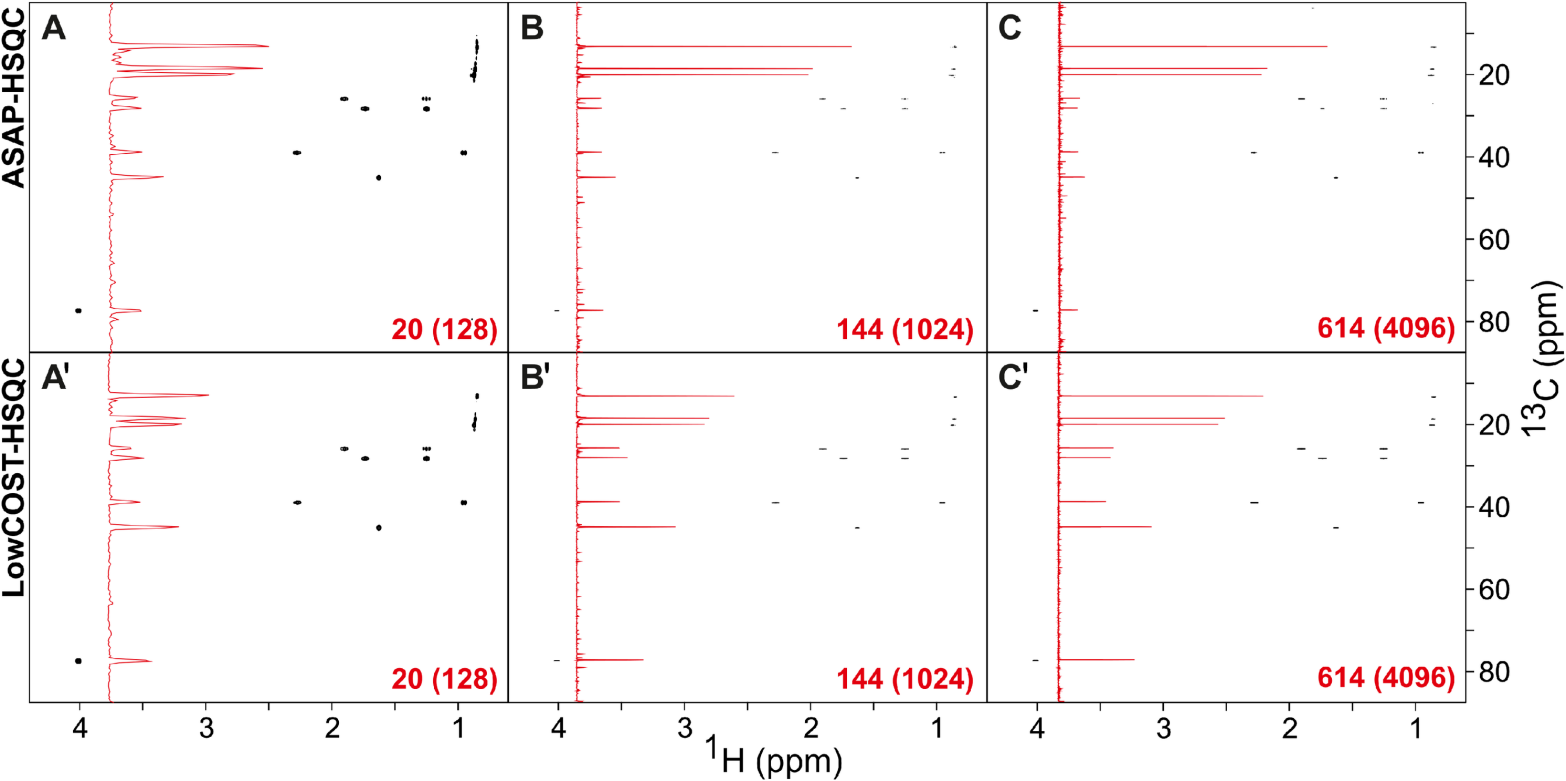
Comparison of ASAP‐HSQC (A–C) and LowCOST‐HSQC (A^*′*^–C^*′*^) spectra for three different 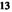
C resolutions acquired on 500‐mM borneol. All spectra were acquired with 512 points in the directly detected dimension, resulting in a 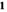
H acquisition time of 112.4 ms. Recovery delays on the order of 100 ms were applied with a fine adjustment of the ASAP‐HSQC delay for the same overall duration. In all cases, 15% NUS was applied, leading to 20(256), 144(1024), and 614(4096) acquired (and NUS‐processed) data points, and to acquisition times of 5 (A,A^*′*^), 39.9 (B,B^*′*^), and 159.8 ms (C,C^*′*^), respectively. After processing with linear prediction and zero filling, 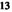
C digital resolutions of 50.1 (A,A^*′*^), 6.26 (B,B^*′*^), and 1.57 Hz (C,C^*′*^) were obtained in overall experiment times of 9 s, 51 s, and 3 min 46 s, respectively. Spectra are displayed with identical settings. The projections along the 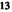
C dimensions are given as inserts in red for each spectrum.

In Figure 2, the resulting effects on sensitivity are measured on the test molecule menthol, which provides a number of different spin system properties. Basic ASAP‐HSQC and LowCOST‐HSQC experiments using broadband‐shaped pulses have been recorded with 15% nonuniform sampling (NUS) and different numbers of $t_{1}$ increments and therefore different acquisition times in the indirect dimension.

We can distinguish two extremes of spin systems for menthol: the methyl groups, for which the signal itself comes from three spins, while only one nearest neighbor atom serves as the main spin reservoir, and all other CH or CH_2_ groups, where one or two recorded protons have a minimum of four directly coupled neighboring spins. All spins have relaxation times in the range of 1–2 s, providing very little recovery between scans within the very short interscan delays $d_{r}$ of ≈100 ms. As expected from theory (see [16] for a thorough theoretical treatment), the three methyl groups (uppermost intensive signals in the spectra of Figure 2) show relatively low intensities in the LowCOST‐HSQC spectra compared with the ASAP‐HSQC. The effect is stronger for short acquisition times and less pronounced for the longest acquisition time applied. Apparently, isotropic mixing spin redistribution has little effect for methyl groups with their small spin reservoir, and the dominating effect of polarization recovery is provided by the Ernst angle–type excitation in the ASAP‐HSQC. Even sensitivity enhancement in the LowCOST‐HSQC cannot counteract this advantage for the ASAP‐HSQC. For long acquisition times, however, the improvement is to some extent outweighed by the reservoir storage along z during $t_{1}$ in the LowCOST‐HSQC, as coupling evolution in the ASAP‐HSQC reduces the reservoir polarization for long $t_{1}$ increments even for the case of a single directly coupled proton.

The situation is very different for all other protons in the other spin systems: The large coupling networks lead to effective redistribution of spin polarization and sensitivity enhancement is sufficient to provide better signal‐to‐noise ratio for the LowCOST‐HSQC as compared with the ASAP‐HSQC. The advantage for the LowCOST‐HSQC significantly increases even for long acquisition time due to the favorable storage of reservoir polarization along z, which has a particularly dramatic effect for the heavily coupled spins.

### 2.3 Constant‐Time LowCOST‐HSQC

**FIGURE 3:**
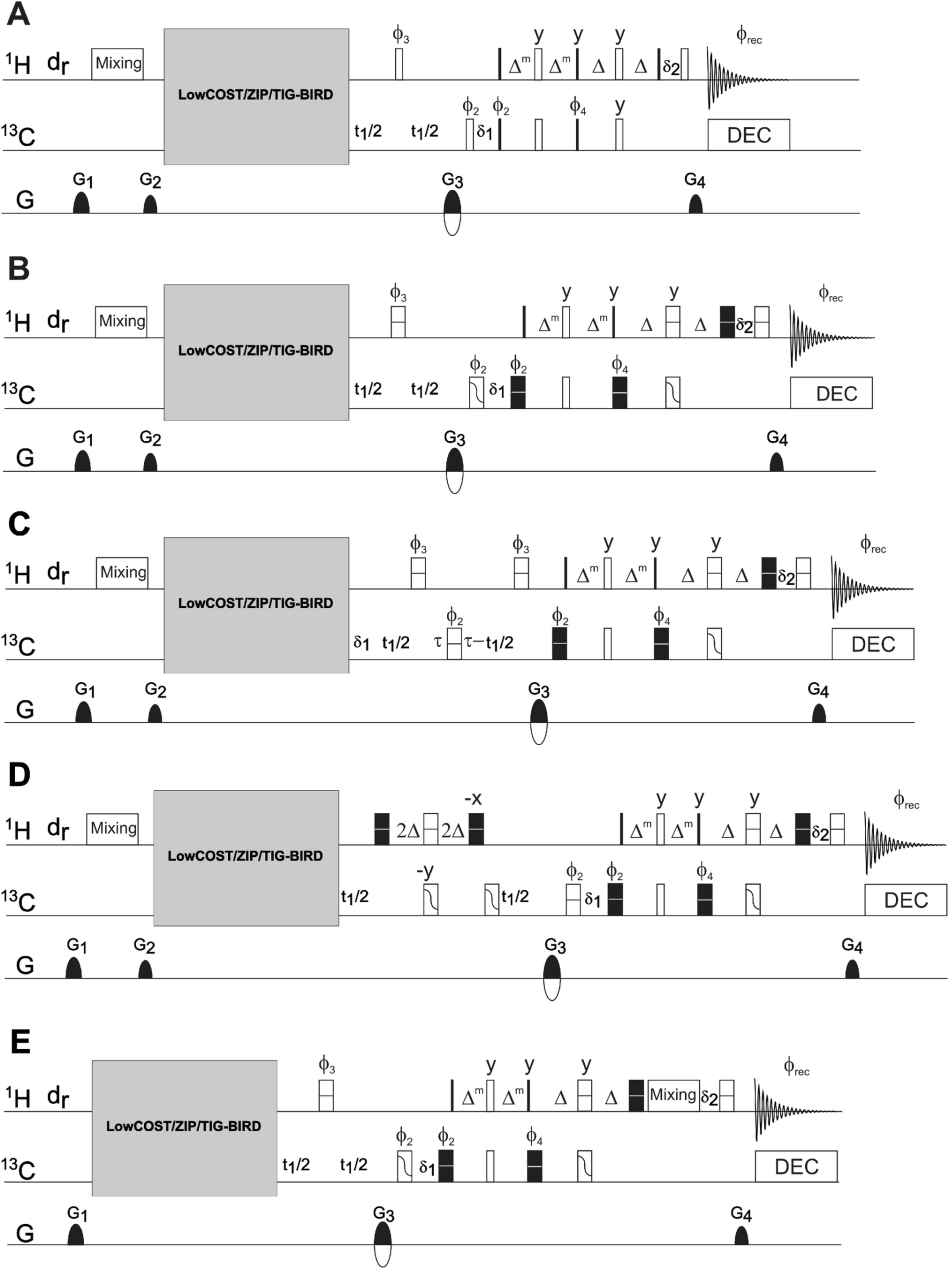
Pulse sequences compilation: Hard pulse LowCOST‐HSQC (A), broadband‐shaped pulse LowCOST‐HSQC (B), constant‐time LowCOST‐HSQC (C), LowCOST‐BIRD 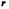
‐HSQC (D), and LowCOST‐HSQC‐TOCSY (E). 90° hard pulses are indicated by thin filled bars, while hard 180° pulses are represented by open bars. All point‐to‐point and universal rotation‐shaped pulses are used as defined in the caption of Figure 1. As J‐compensated pulse sandwiches with a universal rotation 90° pulses are not yet available, corresponding 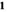
H pulses are applied as hard pulses. For rf‐energy reasons hard 180° pulses are also applied in cases where two simultaneous universal rotation pulses are required, although also J‐compensated BUBU pulse sandwiches [44] may be applied. Big gray boxes indicate the forward‐transfer placeholder, for which one of the transfer elements of Figure 1 has to be filled in. Pulse phases are along x unless indicated otherwise. $\begin{array}{c} \begin{array}{c} \phi_{1}=x,-x,\;\phi_{2}=x,x,-x,-x,\;\phi_{3}=-y,-y,y,y,\;\phi_{4}=-y,-y,-y,-y,y,y, \end{array} \end{array}\begin{array}{c} y,y,\;\phi_{\text{rec}}=x,-x,-x,x \end{array}$. Gradients are $G_{1}=31\%,\;G_{2}=17\%,\;G_{3}=80\%$, and $G_{4}=20.1\%$, where the sign of $G_{3}$ is changed with echo/antiecho selection together with phase $\phi_{5}$. Delays $\delta_{1}$ and $\delta_{2}$ need to be adjusted so that initial $t_{1}$ increments are effectively set to zero and the magnetization is fully refocused before acquisition. DIPSI‐2 is used whenever a mixing period is indicated in the sequences and standard adiabatic ca‐WURST decoupling is applied during acquisition. Recovery delays $d_{r}$ are varied as described with actual acquired spectra.

**FIGURE 4:**
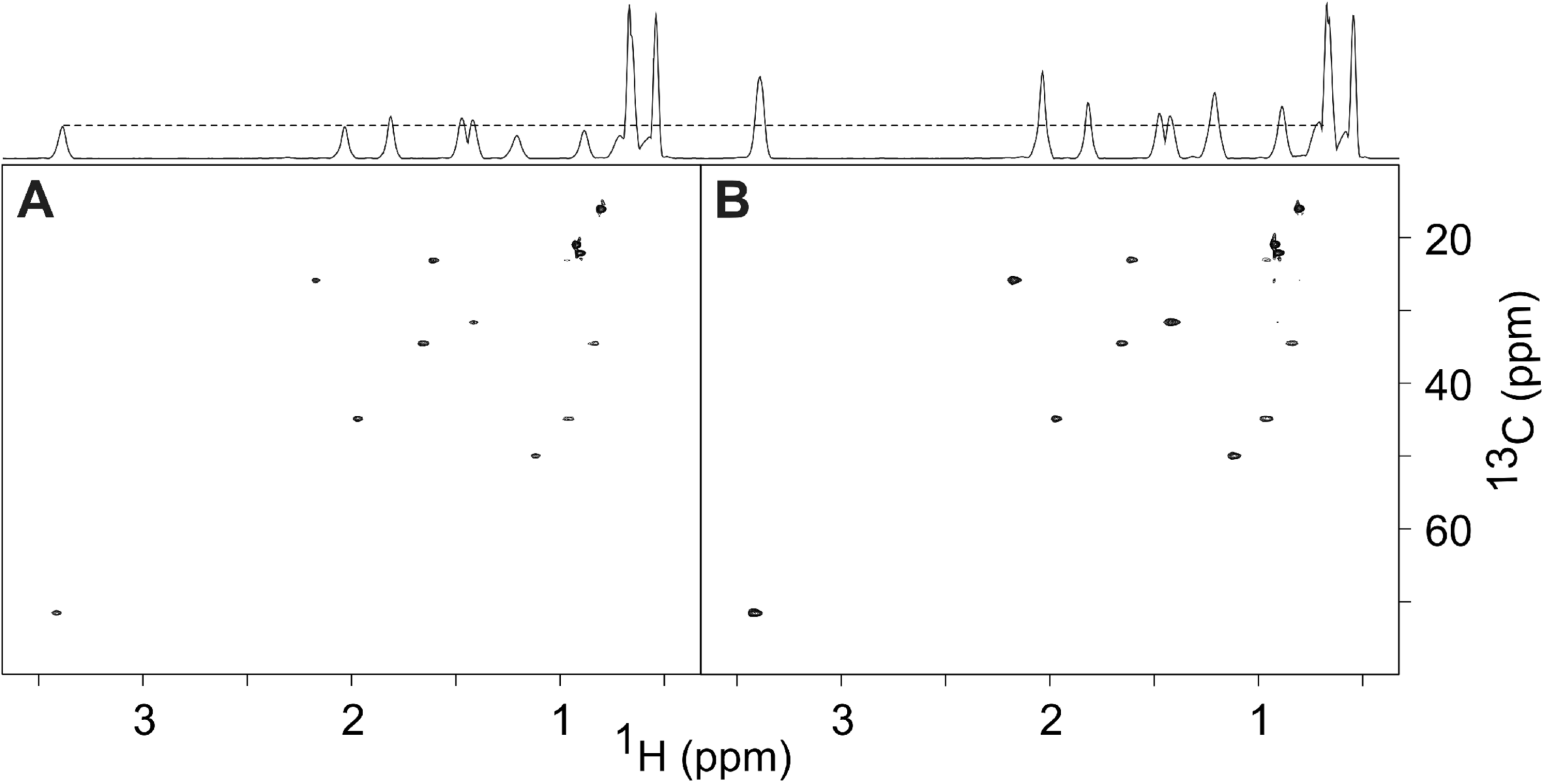
Comparison of two constant‐time LowCOST‐HSQC spectra for demonstrating the effect of isotropic mixing–based redistribution of polarization. Spectra are recorded with a relaxation delay $d_{r}=34.54$ ms (A) and no specified relaxation delay but a DIPSI‐2 isotropic mixing period of equal length and otherwise identical settings (B). The constant‐time delay was set to 2τ=12 ms. Spectra were acquired with 256 ( 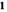
H) × 128 ( 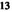
C) points and resulting acquisition times of 63.7 and 6.06 ms, respectively. After processing with linear prediction and zero filling, digital resolution of 3.92 and 41.21 Hz, respectively, was achieved. The overall duration with one scan per increment and four dummy scans was 18 s. Clearly, the improved sensitivity for nonmethyl groups is visible.

In Figure 3C, a constant‐time version of the LowCOST‐HSQC is given. The constant time ensures equal relaxation behavior during $t_{1}$ incrementation, and depending on the overall constant‐time period 2τ, it is able to refocus potential 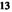
C, 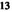
C couplings in fully or partially isotope‐labeled samples. For fully isotope‐labeled small molecules, the LowCOST approach does not make sense, as it relies on detected heteronuclei to be at low abundance. In fluxomics‐type studies, where only local isotope labeling is expected for a small percentage of molecules, the constant‐time LowCOST‐HSQC can be of interest for boosting sensitivity. However, as we do not have access to this type of sample in our laboratory, we can only mention this potential type of application. Instead, we focus on the enhancement in natural abundance samples compared with a conventional constant‐time HSQC with sensitivity enhancement when fast pulsing, that is, short overall experimental times, is required. Example spectra are shown in Figure 4 for menthol. For the applied recovery delay $d_{r}=100$ ms, a clear improvement in intensity for all signals but the isolated methyl groups is found. This improvement can almost exclusively be attributed to the isotropic mixing–based redistribution of spin polarization, which also explains why the methyl groups with their small effective spin systems do not benefit from the LowCOST approach.

### 2.4 LowCOST‐BIRD ‐HSQC

It has been clearly demonstrated in a number of publications [47, 48, 49, 50] that the application of a BIRD 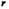 filter element [24, 51] in the center of $t_{1}$ provides a reduced multiplet structure in the indirect dimension that can be highly useful for the measurement of heteronuclear one‐bond couplings. A corresponding LowCOST‐BIRD 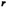
‐HSQC sequence is given in Figure 3D.

**FIGURE 5:**
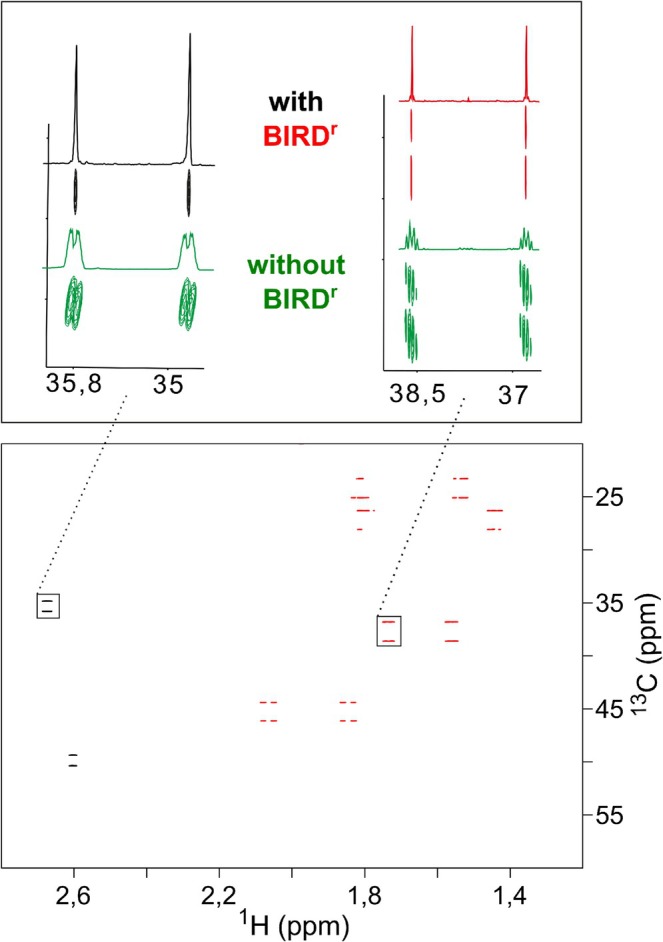
A $\omega_{1}$‐coupled LowCOST‐BIRD 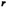
‐HSQC spectrum acquired using sequence Figure 3D (bottom) and the comparison of example cross peaks with a $\omega_{1}$‐coupled LowCOST‐HSQC without BIRD 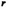 element (top) measured on norcamphor. The BIRD 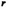 filter element effectively decouples 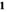
H, 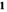
H couplings in the indirect dimension and also acts as a multiplicity editing element in which CH and CH_3_ groups appear with positive cross peaks (black), while CH_2_ groups are inverted (red). In the top part, the reference cross peaks of the $\omega_{1}$‐coupled LowCOST‐HSQC without BIRD 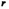 element are shown in green. Clearly, the decoupling effect is visible. Spectra were acquired with 512 ( 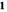
H) × 4096 ( 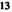
C) points and resulting acquisition times of 266.9 and 339.5 ms, respectively. In addition, linear prediction and zero filling were applied, which results in a final digital resolution of 0.94 ( 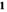
H) and 0.74 Hz ( 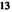
C), respectively. With a recovery delay $d_{r}=100$ ms, 16 dummy scans, and one scan per $t_{1}$ increment, the overall measurement time resulted in 42 min per spectrum.

**FIGURE 6:**
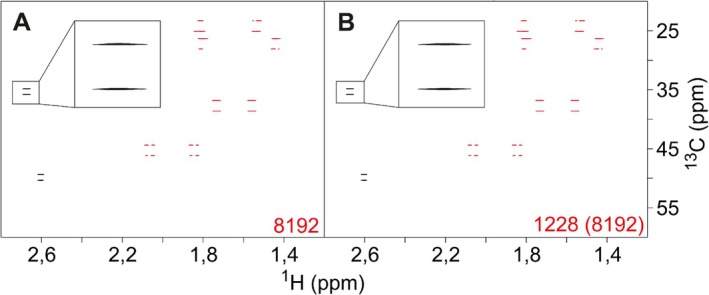
Comparison of $\omega_{1}$‐coupled LowCOST‐BIRD 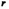
‐HSQC spectra acquired conventionally (A) and using 15% NUS reduction for the indirect dimension (B). The sequence from Figure 3D has been used in both cases using norcamphor as a test compound. Spectra were acquired with 256 ( 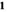
H) × 8192 ( 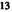
C) points and resulting acquisition times of 133.5 and 679 ms, respectively. Linear prediction and zero filling were applied in both dimensions and in addition to NUS sampling for Spectrum B, which results in a final digital resolution of 1.87 ( 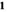
H) and 0.37 Hz ( 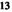
C), respectively. With a recovery delay $d_{r}=50$ ms, 16 dummy scans, and one scan per $t_{1}$ increment, the overall measurement time resulted in 1 h 21 min (A) and 11 min 49 s (B). Spectra are virtually identical.

A comparison of an $\omega_{1}$‐coupled LowCOST‐HSQC and the LowCOST‐BIRD 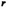
‐HSQC is shown in Figure 5. Clearly, the BIRD 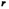
‐enhanced spectrum provides significantly reduced multiplet widths with increased sensitivity and therefore simplifies the determination of one‐bond couplings. However, as coupling measurements have to be performed in the indirect dimension, overall measurement times may become very long due to the large number of necessary $t_{1}$ increments. As a consequence, the LowCOST approach and the application of NUS seems to be particularly favorable. Corresponding example spectra for norcamphor are shown in Figure 6 for conventional acquisition with 8192 increments and for the same resolution acquired with 15% NUS. Previous studies mention that the influence of NUS on the accuracy of coupling measurement is negligible, and although we did not attempt a detailed evaluation of data, a qualitative visual inspection did not show any differences for the two acquisition schemes. The only effect we could observe was a slight expected decrease in sensitivity due to the reduced number of actually recorded increments.

### 2.5 LowCOST‐HSQC‐TOCSY

As has been pointed out for the ASAP‐HSQC‐TOCSY [15], the isotropic mixing sequence does not need to be applied additionally at the beginning of an experiment for efficient and fast redistribution of spin polarization, but if the right type of isotropic mixing multiple pulse sequence is used, it can be combined with a conventional TOCSY period at the end of a 2D acquisition scheme right before the acquisition of individual FIDs. The corresponding pulse sequence for a LowCOST‐HSQC‐TOCSY is provided in Figure 3E. The preferred isotropic mixing case in our laboratory is the classical DIPSI‐2 multiple pulse sequence [52], which guarantees isotropic mixing conditions along all three axes with a bandwidth that roughly corresponds to the applied rf‐amplitude. The latter is a prerequisite for the LowCOST‐HSQC‐TOCSY, as TOCSY transfer with the detected magnetization occurs in the transverse plane, while redistribution of reservoir magnetization happens along z.

**FIGURE 7:**
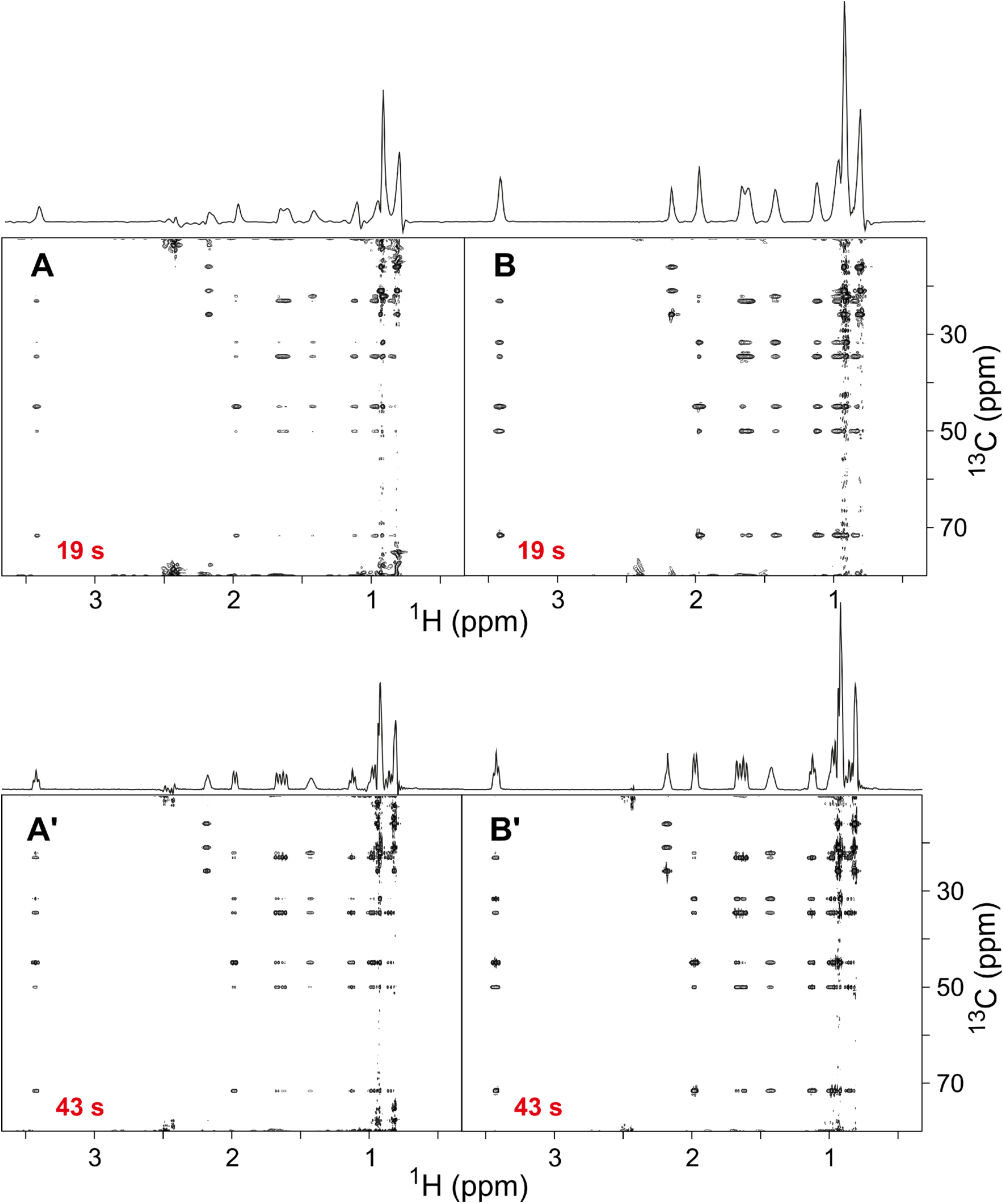
Comparison of conventional and LowCOST‐HSQC‐TOCSY spectra under fast‐pulsing conditions. A conventional TOCSY with sensitivity‐enhanced back transfer [53, 54, 55, 56, 57, 58] (A,A^*′*^), as well as the LowCOST‐HSQC‐TOCSY provided in Figure 3E (B,B^*′*^). The top two spectra (A,B) were acquired with 256 ( 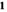
H) × 128 ( 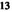
C) points with acquisition times of 63.7 and 6.06 ms, respectively. The recovery delay was set to the shortest possible writing delay on our Bruker spectrometer (30 ms) plus a small correction for the conventional HSQC‐TOCSY to provide exactly identical overall experiment times of 19 s for the two compared spectra. Bottom spectra (A^*′*^,B^*′*^) were acquired with higher resolution in the directly detected dimension with 512 ( 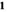
H) × 128 ( 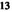
C) points and acquisition times of 127.5 and 6.06 ms, respectively. In addition, the recovery delay was increased by 100 ms compared with the upper spectra. The settings result in exactly identical overall experiment times of 43 s. For isotropic mixing the DIPSI‐2 sequence with a mixing time of 34.54 ms was applied. Data were processed using linear prediction and zero filling in the direct dimension, leading to 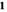
H digital resolutions of 3.92 (A,B) and 1.96 Hz (A^*′*^,B^*′*^), respectively. Positive projections are provided at the top of 2D spectra to indicate relative signal intensities.

Example spectra for a comparison with fast‐pulsing conventional experiments are given in Figure 7. The upper two experiments were acquired with exactly identical overall experiment times of 19 s and are trimmed for minimum measurement time for the experiment. Please be aware that no NUS was applied and 128 increments together with four dummy scans were acquired in 19 s. The recovery delay has been minimized and is determined by technical boundaries like the time needed for disc I/O. Overall acquisition time plus minimum delays add up to an effective recovery time on the order of 100 ms. Under these conditions, the conventional HSQC‐TOCSY provides spectra of remarkable performance, but the LowCOST‐HSQC‐TOCSY shows significantly enhanced sensitivity exceeding a factor of 3 for some resonance. Also, spectral artifacts are reduced. Bottom two spectra have been acquired with longer acquisition times in the direct dimension and an overall effective recovery time on the order of 250 ms. Clearly, the enhanced resolution benefits the spectral quality with a still fast experiment time of 43 s. The improvement in sensitivity of the LowCOST‐TOCSY is comparable.

## 3 Conclusions

Following the original COST‐HSQC designed for 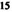
N‐labeled proteins [22], a transfer element that specifically produces $2I_{z}S_{y}$ for heteronuclear coupled IS spins, while maintaining $I_{z}$ polarization for uncoupled I spins, has been developed that allows the storage of unused polarization along z during $t_{1}$ periods and together with sensitivity‐enhanced back transfer its conservation for the following scan. Particularly in fast‐pulsing experiments, this approach guarantees fast repetition rates with isotropic mixing polarization distribution as introduced with the ASAP‐HMQC [10]. Equivalent approaches have been developed independently in the LowCOST‐HSQC [17] and the ZIP‐HSQC [18, 19]. As the LowCOST‐HSQC was reported earlier, we use the term LowCOST‐HSQC throughout this article, while it could also be termed ZIP‐HSQC.

The core of the experiment is the forward‐transfer element, for which three different versions do exist. The original LowCOST version is not really optimized and requires four carbon inversion pulses, while the ZIP element requires only three carbon inversions and the TIG‐BIRD transfer element only two. But in the original LowCOST implementation, already, the use of compensated optimal control–derived pulse shapes has been suggested. The three elements are thoroughly studied here with respect to their transfer properties as hard pulse and broadband compensated shaped pulse versions. Clearly, ZIP and TIG‐BIRD hard pulse versions provide better results than the LowCOST transfer element. Their overall performance, however, is generally bad and the application of hard pulses should only be considered up to 400‐MHz spectrometers. For higher spectrometer fields, the use of shaped pulses is highly recommended. The compensated pulse versions of the three transfer elements essentially provide equivalent results and can be exchange at will in all experiments shown. However, the TIG‐BIRD sequence uses the least amount of pulses and therefore the least amount of rf‐energy, which is beneficial especially in fast‐pulsing experiments. We therefore recommend the offset‐compensated TIG‐BIRD sequence. It should be noted that the supporting information of [19] contains a study that clearly favors the ZIP element over a TIG‐BIRD element. The TIG‐BIRD sequence used ( $45{{}^{\circ}}_{315}-\Delta -180{{}^{\circ}}_{x}-\Delta -45{{}^{\circ}}_{135}$ instead of $45{{}^{\circ}}_{315}-\Delta -180{{}^{\circ}}_{315}-\Delta -90{{}^{\circ}}_{225}$ used here), however, is wrongly derived and must lead to reduced transfer, which also explains the contradicting result.

When comparing ALSOFAST, ASAP, and LowCOST as the most preferential versions of reservoir‐retaining HSQCs for small molecules, four aspects can be identified for the three classes of experiments that influence the resulting sensitivity improvement: (i) Ernst angle–type excitation, which is applied in ALSOFAST/ASAP experiments but is not compatible with the LowCOST approach; (ii) the classical sensitivity‐enhanced back transfer, on the other hand, is used in LowCOST experiments but is not compatible with ALSOFAST/ASAP sequences; (iii) the fast isotropic mixing redistribution of spin polarization is provided in ASAP and LowCOST experiments but not in ALSOFAST‐type approaches; and (iv) the suppression of 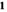
H, 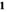
H‐coupling evolution during $t_{1}$ is beneficial in LowCOST experiments but not compatible with either ALSOFAST and ASAP approaches. The approaches are also summarized in Table 1.

**TABLE 1:** Fast‐pulsing HSQC approaches and their enhancement factors.

	ALSOFAST	ASAP	LowCOST/ZIP	Benefitting spins
Ernst angle–type excitation	✓	✓	—	All slow relaxing spins
Sensitivity‐enhanced back transfer	—	—	✓	All multiplicities CH_2_ > CH_3_ > CH
Fast isotropic mixing redistribution	—	✓	✓	Large effective spin systems
Magn. reservoir along z during $t_{1}$	—	—	✓	Spins with large multiplets
Best overall performance	Isolated spins only	Short overall experiment times	Long $\omega_{1}$ acquisition times	

This leads to classification regarding the expected benefits for the different approaches: The ALSOFAST approach is only favorable for isolated spins for which the Ernst angle–type excitation is the only way to improve sensitivity. The ASAP‐HSQC combines the Ernst angle–type excitation with fast isotropic mixing polarization redistribution, leading to best results for small spin systems with relatively little reservoir and short acquisition times in the indirect dimension. The LowCOST approach increases its performance with the number of directly coupled and even more with the applied acquisition time in the indirect dimension. As such, LowCOST‐type experiments are clear favorites for experiments that target utmost resolution in the indirect dimension, while very short experiments with very few $t_{1}$ increments are preferably run as ASAP‐type experiments.

The constant‐time LowCOST‐HSQC, LowCOST‐BIRD 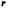
‐HSQC, and LowCOST‐HSQC‐TOCSY experiments provided here are the logic extensions for fast‐pulsing heteronuclear correlation experiments provided in their ALSOFAST/ASAP version in [13, 14, 15].

## Conflicts of Interest

The authors declare no conflicts of interest.

## Data Availability

Original NMR data, pulses, pulse sequences, and code for simulations are available in [59]. A temporary link for review is available via https://www.radar‐service.eu/radar/de/dataset/p0a0f9g3vwnefw6f?token=zXtNEzdIsyMGWZpiHtZa.

## References

[1] A. Ross , M. Salzmann , and H. Senn , “Fast‐HMQC Using Ernst Angle Pulses: An Efficient Tool for Screening of Ligand Binding to Target Proteins,” Journal of Biomolecular NMR 10, no. 4 (1997): 389–396. 20859783 10.1023/A:1018361214472

[2] P. Schanda , E. Kupče , and B. Brutscher , “SOFAST‐HMQC Experiments for Recording Two‐Dimensional Deteronuclear Correlation Spectra of Proteins Within a Few Seconds,” Journal of Biomolecular NMR 33, no. 4 (2005): 199–211. 16341750 10.1007/s10858-005-4425-x

[3] P. Schanda and B. Brutscher , “Very Fast Two‐Dimensional NMR Spectroscopy for Real‐Time Investigation of Dynamic Events in Proteins on the Time Scale of Seconds,” Journal of the American Chemical Society 127, no. 22 (2005): 8014–8015. 15926816 10.1021/ja051306e

[4] P. Schanda , H. Van Melckebeke , and B. Brutscher , “Speeding Up Three‐Dimensional Protein NMR Experiments to a Few Minutes,” Journal of the American Chemical Society 128, no. 28 (2006): 9042–9043. 16834371 10.1021/ja062025p

[5] E. Lescop , P. Schanda , and B. Brutscher , “A set of BEST Triple‐Resonance Experiments for Time‐Optimized Protein Resonance Assignment,” Journal of Magnetic Resonance 187, no. 1 (2007): 163–169. 17468025 10.1016/j.jmr.2007.04.002

[6] J. Farjon , J. Boisbouvier , P. Schanda , A. Pardi , J.‐P. Simorre , and B. Brutscher , “Longitudinal‐Relaxation‐Enhanced NMR Experiments for the Study of Nucleic Acids in Solution,” Journal of the American Chemical Society 131, no. 24 (2009): 8571–8577. 19485365 10.1021/ja901633y PMC2846706

[7] R. R. Ernst and W. A. Anderson , “Application of Fourier Transform Spectroscopy to Magnetic Resonance,” Review of Scientific Instruments 37, no. 1 (1966): 93–102.

[8] R. Ernst , “Sensitivity Enhancement in Magnetic Resonance,” Advances in Magnetic and Optical Resonance, ed. J. S. Waugh (Academic Press, 1966), 1–135.

[9] L. Mueller , “Alternate HMQC Experiments for Recording HN and HC‐Correlation Spectra in Proteins at High Throughput,” Journal of Biomolecular NMR 42, no. 2 (2008): 129–137. 18820839 10.1007/s10858-008-9270-2

[10] E. Kupče and R. Freeman , “Fast Multidimensional NMR by Polarization Sharing,” Magnetic Resonance in Chemistry 45, no. 1 (2007): 2–4. 17125135 10.1002/mrc.1931

[11] J. Furrer , “A Robust, Sensitive, and Versatile HMBC Experiment for Rapid Structure Elucidation by NMR: IMPACT‐HMBC,” Chemical Communications 46, no. 19 (2010): 3396–3398. 20358135 10.1039/c000964d

[12] D. Schulze‐Sünninghausen , J. Becker , and B. Luy , “Rapid Heteronuclear Single Quantum Correlation NMR Spectra at Natural Abundance,” Journal of the American Chemical Society 136, no. 4 (2014): 1242–1245. 24417402 10.1021/ja411588d

[13] D. Schulze‐Sünninghausen , J. Becker , M. R. M. Koos , and B. Luy , “Improvements, Extensions, and Practical Aspects of Rapid ASAP‐HSQC and ALSOFAST‐HSQC Pulse Sequences for Studying Small Molecules at Natural Abundance,” Journal of Magnetic Resonance 281 (2017): 151–161. 28603039 10.1016/j.jmr.2017.05.012

[14] J. Becker and B. Luy , “CLIP–ASAP‐HSQC for Fast and Accurate Extraction of One‐Bond Couplings From Isotropic and Partially Aligned Molecules,” Magnetic Resonance in Chemistry 53, no. 11 (2015): 878–885. 26137959 10.1002/mrc.4276

[15] J. Becker , M. R. M. Koos , D. Schulze‐Sünninghausen , and B. Luy , “ASAP‐HSQC‐TOCSY for Fast Spin System Identification and Extraction of Long‐Range Couplings,” Journal of Magnetic Resonance 300 (2019): 76–83. 30711785 10.1016/j.jmr.2018.12.021

[16] M. R. M. Koos and B. Luy , “Polarization Recovery During ASAP and SOFAST/ALSOFAST‐Type Experiments,” Journal of Magnetic Resonance 300 (2019): 61–75. 30711784 10.1016/j.jmr.2018.12.014

[17] D. Schulze‐Sünninghausen , “Entwicklung und Optimierung Schneller Mehrdimensionaler NMR‐Experimente,” (PhD Thesis, Karlsruher Institut für Technologie, 2016).

[18] E. Kupče , L. Frydman , A. G. Webb , J. R. J. Yong , and T. D. W. Claridge , “Parallel Nuclear Magnetic Resonance Spectroscopy,” Nature Reviews Methods Primers 1, no. 1 (2021): 1–23.

[19] J. R. J. Yong , A. L. Hansen , E. Kupče , and T. D. W. Claridge , “Increasing Sensitivity and Versatility in NMR Supersequences With New HSQC‐Based Modules,” Journal of Magnetic Resonance 329 (2021): 107027. 34246882 10.1016/j.jmr.2021.107027

[20] J. Briand and O. W. Sørensen , “A Novel Pulse Sequence Element for Biselective and Independent Rotations With Arbitrary Flip Angles and Phases for I and I{S} Spin Systems,” Journal of Magnetic Resonance 125, no. 1 (1997): 202–206. 9245382 10.1006/jmre.1996.1095

[21] J. Briand and O. W. Sørensen , “Simultaneous and Independent Rotations With Arbitrary Flip Angles and Phases for I, IS^α^, and IS^β^ Spin Systems,” Journal of Magnetic Resonance 135, no. 1 (1998): 44–49. 9799673 10.1006/jmre.1998.1556

[22] M. Deschamps and I. D. Campbell , “Cooling Overall Spin Temperature: Protein NMR Experiments Optimized for Longitudinal Relaxation Effects,” Journal of Magnetic Resonance 178, no. 2 (2006): 206–211. 16249110 10.1016/j.jmr.2005.09.011

[23] K. Takegoshi , K. Ogura , and K. Hikichi , “A Perfect Spin Echo in a Weakly Homonuclear J‐Coupled Two Spin‐System,” Journal of Magnetic Resonance 84, no. 3 (1989): 611–615.

[24] J. R. Garbow , D. P. Weitekamp , and A. Pines , “Bilinear Rotation Decoupling of Homonuclear Scalar Interactions,” Chemical Physics Letters 93, no. 5 (1982): 504–509.

[25] S. Wimperis and R. Freeman , “An Excitation Sequence Which Discriminates Between Direct and Long‐Range CH Coupling,” Journal of Magnetic Resonance 58, no. 2 (1984): 348–353.

[26] O. W. Sørensen , “Selective Rotations Using Non‐Selective Pulses and Heteronuclear Couplings,” Bulletin of Magnetic Resonance 16 (1994): 49–53.

[27] Y. T. Woordes , T. Reinsperger , S. Ehni , and B. Luy , “Robust Bilinear Rotations,” Science Advances 11, no. 35 (2025): eadx7094. 40880465 10.1126/sciadv.adx7094 PMC12396312

[28] Y. T. Woordes and B. Luy , “Robust Bilinear Rotations II,” Magnetic Resonance Discussions 2025 (2025): 1–20. 10.5194/mr-7-1-2026 PMC12919721 41727561

[29] T. E. Skinner , T. O. Reiss , B. Luy , N. Khaneja , and S. J. Glaser , “Application of Optimal Control Theory to the Design of Broadband Excitation Pulses for High‐Resolution NMR,” Journal of Magnetic Resonance 163, no. 1 (2003): 8–15. 12852902 10.1016/s1090-7807(03)00153-8

[30] K. Kobzar , T. E. Skinner , N. Khaneja , S. J. Glaser , and B. Luy , “Exploring the Limits of Broadband Excitation and Inversion Pulses,” Journal of Magnetic Resonance 170, no. 2 (2004): 236–243. 15388086 10.1016/j.jmr.2004.06.017

[31] T. E. Skinner , T. O. Reiss , B. Luy , N. Khaneja , and S. J. Glaser , “Reducing the Duration of Broadband Excitation Pulses Using Optimal Control With Limited RF Amplitude,” Journal of Magnetic Resonance 167, no. 1 (2004): 68–74. 14987600 10.1016/j.jmr.2003.12.001

[32] K. Kobzar , B. Luy , N. Khaneja , and S. J. Glaser , “Pattern Pulses: Design of Arbitrary Excitation Profiles as a Function of Pulse Amplitude and Offset,” Journal of Magnetic Resonance 173, no. 2 (2005): 229–235. 15780915 10.1016/j.jmr.2004.12.005

[33] K. Kobzar , T. E. Skinner , N. Khaneja , S. J. Glaser , and B. Luy , “Exploring the Limits of Broadband Excitation and Inversion: II. Rf‐Power Optimized Pulses,” Journal of Magnetic Resonance 194, no. 1 (2008): 58–66. 18586540 10.1016/j.jmr.2008.05.023

[34] K. Kobzar , S. Ehni , T. E. Skinner , S. J. Glaser , and B. Luy , “Exploring the Limits of Broadband 90° and 180° Universal Rotation Pulses,” Journal of Magnetic Resonance 225 (2012): 142–160. 23142001 10.1016/j.jmr.2012.09.013

[35] T. E. Skinner , N. I. Gershenzon , M. Nimbalkar , W. Bermel , B. Luy , and S. J. Glaser , “New Strategies for Designing Robust Universal Rotation Pulses: Application to Broadband Refocusing at Low Power,” Journal of Magnetic Resonance 216 (2012): 78–87. 22325853 10.1016/j.jmr.2012.01.005

[36] M. R. M. Koos , H. Feyrer , and B. Luy , “Broadband Excitation Pulses With Variable RF Amplitude‐Dependent Flip Angle (RADFA),” Magnetic Resonance in Chemistry 53, no. 11 (2015): 886–893. 26259565 10.1002/mrc.4297

[37] J. D. Haller , D. L. Goodwin , and B. Luy , “SORDOR Pulses: Expansion of the Böhlen–Bodenhausen Scheme for Low‐Power Broadband Magnetic Resonance,” Magnetic Resonance 3, no. 1 (2022): 53–63. 37905174 10.5194/mr-3-53-2022 PMC10539771

[38] S. Slad , W. Bermel , R. Kümmerle , D. Mathieu , and B. Luy , “Band‐Selective Universal 90° and 180° Rotation Pulses Covering the Aliphatic Carbon Chemical Shift Range for Triple Resonance Experiments on 1.2 GHz Spectrometers,” Journal of Biomolecular NMR 76, no. 5‐6 (2022): 185–195. 36418752 10.1007/s10858-022-00404-1 PMC9712393

[39] D. Joseph and C. Griesinger , “Optimal Control Pulses for the 1.2‐GHz (28.2‐T) NMR Spectrometers,” Science Advances 9, no. 45 (2023): eadj1133. 37948513 10.1126/sciadv.adj1133 PMC10637738

[40] C. J. Buchanan , G. Bhole , G. Karunanithy , et al., “Seedless: On‐the‐Fly Pulse Calculation for NMR Experiments,” Nature Communications 16, no. 1 (2025): 7276. 10.1038/s41467-025-61663-8 PMC12332029 40775231

[41] S. Slad and B. Luy , “Single Spin Exact Gradients for the Optimization of Complex Pulses and Pulse Sequences,” Journal of Biomolecular NMR 80 (2026): 6, 10.1007/s10858-025-00486-7. 41701381 PMC12913341

[42] Y. T. Woordes , K. Kobzar , S. Ehni , et al., “Ultrabroadband 1D and 2D NMR Spectroscopy,” Angewandte Chemie International Edition 138 (2025): e15467. 10.1002/anie.202515467 PMC12790362 41307299

[43] S. Ehni and B. Luy , “BEBEtr and BUBI: J‐Compensated Concurrent Shaped Pulses for 1H–13C Experiments,” Journal of Magnetic Resonance 232 (2013): 7–17. 23673080 10.1016/j.jmr.2013.04.007

[44] S. Ehni , M. R. M. Koos , T. Reinsperger , J. D. Haller , D. L. Goodwin , and B. Luy , “Concurrent J‐Evolving Refocusing Pulses,” Journal of Magnetic Resonance 336 (2022): 107152. 35189510 10.1016/j.jmr.2022.107152

[45] B. Luy , K. Kobzar , T. E. Skinner , N. Khaneja , and S. J. Glaser , “Construction of Universal Rotations From Point‐to‐Point Transformations,” Journal of Magnetic Resonance 176, no. 2 (2005): 179–186. 16009584 10.1016/j.jmr.2005.06.002

[46] Y. T. Woordes and B. Luy , “Broadband INADEQUATE Experiments,” to be submitted.

[47] K. Fehér , S. Berger , and K. E. Kövér , “Accurate Determination of Small One‐Bond Heteronuclear Residual Dipolar Couplings by F1 Coupled HSQC Modified With a G‐BIRD^(r)^ Module,” Journal of Magnetic Resonance 163, no. 2 (2003): 340–346. 12914850 10.1016/s1090-7807(03)00113-7

[48] C. M. Thiele and W. Bermel , “Speeding Up the Measurement of One‐Bond Scalar (^1^ *J*) and Residual Dipolar Couplings (^1^ *D*) by using Non‐Uniform Sampling (NUS),” Journal of Magnetic Resonance 216 (2012): 134–143. 22342269 10.1016/j.jmr.2012.01.008

[49] L. Kaltschnee , A. Kolmer , I. Timári , et al., ““Perfecting” Pure Shift HSQC: Full Homodecoupling for Accurate and Precise Determination of Heteronuclear Couplings,” Chemical Communications 50, no. 99 (2014): 15702–15705. 25360807 10.1039/c4cc04217d

[50] J. Furrer , M. John , H. Kessler , and B. Luy , “J‐Spectroscopy in the Presence of Residual Dipolar Couplings: Determination of One‐Bond Coupling Constants and Scalable Resolution,” Journal of Biomolecular NMR 37, no. 3 (2007): 231–243. 17235497 10.1007/s10858-006-9130-x

[51] D. Uhrín , T. Liptaj , and K. E. Kövér , “Modified BIRD Pulses and Design of Heteronuclear Pulse Sequences,” Journal of Magnetic Resonance 101, no. 1 (1993): 41–46.

[52] A. J. Shaka , C. J. Lee , and A. Pines , “Iterative Schemes for Bilinear Operators; Application to Spin Decoupling,” Journal of Magnetic Resonance 77, no. 2 (1988): 274–293.

[53] L. Lerner and A. Bax , “Sensitivity‐Enhanced Two‐Dimensional Heteronuclear Relayed Coherence Transfer NMR Spectroscopy,” Journal of Magnetic Resonance 69, no. 2 (1986): 375–380.

[54] G. Mackin and A. J. Shaka , “Phase‐Sensitive Two‐Dimensional HMQC and HMQC‐TOCSY Spectra Obtained Using Double Pulsed‐Field‐Gradient Spin Echoes,” Journal of Magnetic Resonance A 118, no. 2 (1996): 247–255.

[55] K. E. Kövér , V. J. Hruby , and D. Uhrín , “Sensitivity‐ and Gradient‐Enhanced Heteronuclear Coupled/Decoupled HSQC–TOCSY Experiments for Measuring Long‐Range Heteronuclear Coupling Constants,” Journal of Magnetic Resonance 129, no. 2 (1997): 125–129. 9441876 10.1006/jmre.1997.1265

[56] W. Koźmiński , “Simplified Multiplet Pattern HSQC‐TOCSY Experiment for Accurate Determination of Long‐Range Heteronuclear Coupling Constants,” Journal of Magnetic Resonance 137, no. 2 (1999): 408–412. 10089176 10.1006/jmre.1998.1700

[57] B. L. Marquéz , W. H. Gerwick , and R. Thomas Williamson , “Survey of NMR Experiments for the Determination of ^ *n* ^ *J*(C,H) Heteronuclear Coupling Constants in Small Molecules,” Magnetic Resonance in Chemistry 39, no. 9 (2001): 499–530.

[58] K. Kobzar and B. Luy , “Analyses, Extensions and Comparison of Three Experimental Schemes for Measuring (nJCH+DCH)‐Couplings at Natural Abundance,” Journal of Magnetic Resonance 186, no. 1 (2007): 131–141. 17336556 10.1016/j.jmr.2007.02.005

[59] D. Schulze‐Sünninghausen , J. Becker , M. R. M. Koos , and B. Luy , LowCOST‐HSQC Variants for Fast Pulsing High $\omega_{1}$‐Resolved 2D‐Experiments [Dataset], (2025), 10.35097/p0a0f9g3vwnefw6f. PMC13238328 41989383

